# Evaluation of the cognitive, physiological, and biomarker effects of heavy metal exposure in Wistar rats

**DOI:** 10.14202/vetworld.2024.1855-1863

**Published:** 2024-08-24

**Authors:** Senna Mukhi, Poornima Ajay Manjrekar, Rukmini Mysore Srikantiah, Sindhu Harish, Himani Kotian, Y. Lakshmisha Rao, Anita Sherly

**Affiliations:** 1 Department of Biochemistry, Kasturba Medical College, Mangalore, Manipal Academy of Higher Education, Manipal, Karnataka, India; 2 Department of Community Medicine, Kasturba Medical College, Mangalore, Manipal Academy of Higher Education, Manipal, Karnataka, India; 3 Department of Anatomy, Kasturba Medical College, Mangalore, Manipal Academy of Higher Education, Manipal, Karnataka, India

**Keywords:** arsenic, mercury, vanadium, Wistar rats

## Abstract

**Background and Aim:**

Individuals exposed to heavy metals are known to experience physiological and biochemical changes, which raise questions regarding possible health effects. In our earlier research, significant concentrations of vanadium (V), mercury (Hg), cadmium (Cd), and arsenic (As) were found in food and medical packaging materials. This study aimed to evaluate the cognitive, physiological, and biomarker effects of select heavy metal exposure in Wistar rats.

**Materials and Methods:**

Over a 13-week period, five groups of rats (six rats per group, with both males and females) were assessed to study the effects of oral exposure to V, Hg, Cd, and As. The study focused on evaluating physiological, cognitive, and biochemical markers, with the results compared to those of a control group.

**Results:**

Comparing all groups of rats treated with heavy metals, the study revealed significant deficits in learning and spatial orientation (water maze test); rats treated with V, Cd, and Hg showed signs of depression. Rats treated with As also showed signs of hyperactivity, which may indicate a connection to attention-deficit hyperactivity disorder (rat tail suspension test). The groups exposed to different heavy metals varied in their physiological (water and food intake, urine and feces output) and biochemical responses (enzyme-linked immunosorbent assay, prostate-specific antigen, T3, T4, thyroid-stimulating hormone, carcinoembryonic antigen, and blood glucose analysis), with Hg exhibiting the strongest impacts. Rats given Hg showed signs of hypothyroidism, such as increased food intake and weight gain.

**Conclusion:**

This study clarifies the complex relationships between exposure to heavy metals and various biological systems, shedding light on their potential health impacts. The findings provide insight into the effects of heavy metals on neural and thyroid tissues, as well as their propensity to cause cellular dedifferentiation. However, the study has certain limitations, such as the relatively short duration of exposure and the use of only a few selected biomarkers. Future research should focus on long-term exposure studies, incorporate a broader range of biomarkers, and explore the underlying mechanisms at a molecular level to better understand the full spectrum of health risks associated with heavy metal exposure.

## Introduction

The possible health hazards associated with heavy metals, such as vanadium (V), cadmium (Cd), arsenic (As), and mercury (Hg), are a major issue when they are found in food and drug packaging. When consumed, these metals can leach into consumables, potentially harmful to human health [1]. Transition metal V, which is frequently used in alloys, is found in some packaging materials [2]. Cd is often used in stabilizers and pigments as a coating and plasticizer in packaging materials [3]. As is present in dyes and pigments of the textiles, non-stick coated pans, or even ceramics [4]. Although not a direct constituent of materials, Hg is an important element in industrial machinery used in manufacturing processes and often finds itself as a contaminant in packaging materials [5].

The transfer of heavy metals from packaging materials to packaged constituents may occur due to direct contact and may be enhanced by the effects of moisture, temperature, pH, time of contact, etc. [6]. Given the huge market for these materials in the present day, the consumption of these materials in both the food and drug packaging industries is expected. It is, therefore, imperative to study the effects of these metals on health. The effects of heavy metals in humans include reproductive complications, neurotoxicity, organ damage, and cancer in subjects exposed to these metals over a period of time [7]. The imperceptible leaching of heavy metals into packaged items and their consumption remains to be explored. When heavy metals such as V, Cd, Hg, and As seep into food through different processes during manufacturing or from packaging materials, it can seriously endanger human health. Exposure to V has been linked to gastrointestinal and respiratory problems, whereas Cd is a recognized carcinogen that negatively impacts the kidneys and lungs [8–10]. Hg can be harmful to the nervous system, especially in developing fetuses and young children [11]. Strong toxins and carcinogens, such as As, can damage many organ systems and have been linked to a number of cancers [12, 13]. Previous studies showed the deposition of heavy metals in tissues such as the brain, thyroid, and gonads and are most susceptible to any damage. Hence, the study was undertaken to understand the functional incapacities of the organs upon exposure to heavy metals [14]. In continuation of our previous study [15], this study examines the amount of heavy metals found in packaging materials and their effect on Wistar strain rats on cognitive, biochemical, and physiological parameters.

## Materials and Methods

### Ethical approval

This study was approved by the Institutional Animal Ethics Committee of Kasturba Medical College, Mangalore (KMC/MNG/IAEC/09/2023). This study was conducted in accordance with the ARRIVE guidelines [16]. Animals were sacrificed by an overdose of ether (20% of their body weight).

### Study period and location

The study period was for 13 weeks (June to September-2023) and was conducted at Department of Biochemistry, Central Animal House, Bejai, Kasturba Medical College, Mangalore.

### Standard heavy metals

Heavy metal standards were procured from the Nanoshel Group of Companies, Intelligent Materials, Pvt. Ltd., Punjab, India. V pentoxide, As trioxide, Cd acetate, and laboratory-grade mercuric chloride were in the purity range of 98.5%–99.9%.

### Experimental animals

Twenty-one-day weanlings of Wistar strain rats of either sex were bred and procured from Central Animal House, KMC, Mangalore, and were chosen for the study and housed in separate cages with 2 animals in each cage of the same gender. Animals were fed regular rat chow (commercially available organic rat chow procured from Champaka Feeds and Foods, Bangalore, India) and water. A 12-h dark and light cycle was maintained along with a room temperature of 25°C (±2°C) in accordance with Committee for the Purpose of Control and Supervision of Experiments on Animals guidelines [17]. Animals were acclimatized for 2 weeks, by which time they attained a weight of 100–120 g, after which they were included in the study.

### Dosage and route of administration

The lethal dose 50 (LD_50_) values for individual heavy metals were tested as per The Organization for Economic Cooperation and Development guidelines (*Up-and-Down Procedure*) using 8-week-old nulliparous and non-pregnant female rats [18]. Briefly, after an overnight fast during which food but not water was withheld, the test substance was given to the animals through gavage in a single dose during the experimental procedures. The animals were weighed before dosing, and the dose was determined by measuring each animal’s individual body weight. Food was not allowed for an extra 3–4 h after the drug was administered. One animal was initially dosed in the study’s limit test at 2000 mg/kg. Four more animals received doses one after the other if the animal survived [19]. We calculated the lethal doses; The L_D5_0 for V was 84 mg/kg, Hg 15 mg/kg, As 40 mg/kg, and Cd 233 mg/kg.

### Study groups

Wistar strain rats 100–120 *g* were grouped as follows. The total number of rats were 6 rats per group (3 males and 3 females) = 30 rats.

- Group 1: Control group, untreated (1 mL of water)
- Group 2: V treated (16.8 mg/kg)
- Group 3: Hg treated (3 mg/kg)
- Group 4: As treated (8 mg/kg)
- Group 5: Cd treated (46.6 mg/kg).

### Preparation of doses

Based on the LD_50_ values, it was decided to use the test doses and groups as mentioned above. One-twentieth of the LD_50_ dose was administered to the rats to assess chronic toxicity. To create a stock solution containing 100 μg of heavy metals per mL, each salt was dissolved in distilled water. The dose was prepared at each time treatment was administered to the rats.

### Duration of the study

**Figure-1:**
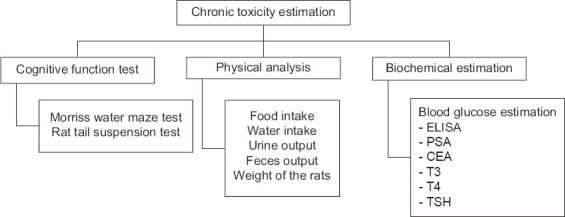
The flow chart of methodology.

The study was lasted for 13 weeks, where the heavy metal salts were dosed every alternate day by oral gavage with *ad libitum* access to food and water. The flow chart of the methodology is highlighted in Figure-1.

### Cognitive function tests

Morris’s water maze and rat tail suspension tests were conducted as part of cognitive and behavioral studies.

#### Morris water maze test

Spatial learning and memory were evaluated using the Morris water maze test. The method was followed as described by Vangalapati *et al*. [20]. Testing was started on the 90^th^ day after treatment and continued for 3 days. A sizable water pool was filled with a submerged platform. Animals were trained on a visible platform on days 1 and 2 for a hidden platform. On day 3, a final trial was conducted to test spatial memory by recording the time spent in the target quadrant (Time to platform- TTP), time spent to reach the target quadrant, and time spent in the original quadrant. The test was performed in triplicate. The average of the 3 values was taken as the test result.

#### Rat tail suspension test

On the 94^th^ day following treatment, a tail suspension test was conducted. Rats were suspended by their tails and allowed to dangle freely as part of the rat tail suspension test, an established technique for evaluating behaviors resembling depression and methodology as described by Zhai *et al*. [21]. The time of mobility and immobility of rats was recorded in seconds. Mice were deemed immobile when they gave up trying to flee and hung by their tails motionless. The test was performed in triplicates. The average of the 3 values was taken as the test result.

### Physiological tests

#### Food and water intake

Water and rat chow were weighed and placed in the cage for consumption. The total water and chow consumed over a period of 13 *week*s were recorded in grams (g).

#### Rat weight

The weight of the rats was monitored weekly. Each rat was weighed using a precise weighing balance before administering the dose, with measurements recorded to the closest gram.

#### Quantification of urine and feces

During the 12^th^ week of the study, each rat was individually placed in the metabolic cage for 12 h to collect and quantify their urine and feces [22].

### Biochemical investigations

#### Blood glucose estimation by glucometer

Blood samples were collected by puncturing the lateral tail vein with a sterile 23 gauze needle. The test was performed after an overnight fast of 8–12 h using a glucometer and commercially available test strips (Accuchek, India; Procured from Durga Laboratory, Mangalore, Karnataka, India) [23].

#### Enzyme-linked immunosorbent assay (ELISA) tests

Prostate-specific antigen (PSA), carcinoembryonic antigen (CEA), and thyroid function tests (T3, T4, thyroid-stimulating hormone [TSH]) were estimated in the serum samples using commercially available ELISA kits procured from Origin Diagnostics, Kerala, India, on an ELX-800 ELISA plate reader (Agilent Technologies, U.S.A).

### Sacrificing the rats

All rats were sacrificed by an overdose of ether (20% of their body weight) [24], blood (3 mL) was collected by cardiac puncture, and the collected blood was centrifuged at 1278× *g* for 15 min. The serum was aliquoted and stored at −20°C for the above tests. The carcass was discarded in accordance with biosafety standards (Ramky Energy and Environment Ltd, Mangalore, India).

### Statistical analysis

All the tests were performed in triplicates (cognitive function tests), mean ± standard deviation of all the other parameters were noted in Microsoft Excel 2021 (Microsoft Office, Washington, USA), and the normality of the data was checked using the Shapiro–Wilk test, and accordingly, Student’s t-test or the Mann–Whitney U test was used to evaluate any significant difference. These statistical tests were conducted using Jamovi (3^rd^ generation, version 2.4.11; https://www.jamovi.org).

## Results

### Cognitive function tests

#### Morris water maze test

The escape latency and time spent in the target and original quadrant were quantified in seconds (s) for all five groups and are presented in Table-1.

**Table-1:** Cognitive function tests in rats treated with select heavy metals.

Cognitive function test	Groups/Parameters	Control	Vanadium		Cadmium		Arsenic		Mercury	
				
		Mean ± SD	Mean ± SD	p-value	Mean ± SD	p-value	Mean ± SD	p-value	Mean ± SD	p-value
Morris water maze test	Escape latency (s)	10.5 ± 3.2	35.8 ± 12	< 0.001*	15.3 ± 5.2	0.08	9.17 ± 2.9	0.47	74.3 ± 23.0	< 0.001
	Time spent in original quadrant (s)	11.7 ± 1.7	16.2 ± 2.9	0.009	18.5 ± 3.9	0.003	4.17 ± 1.1	< 0.001	19.3 ± 4.68	0.004
	Time spent target quadrant (s)	47.7 ± 5.3	21.2 ± 9	< 0.001	16 ± 3.6	< 0.001	3.83 ± 1.4	< 0.001	24.2 ± 13.1	0.003
Tail suspension test	Seconds of mobility	219 ± 28	136 ± 52	0.007	135 ± 16	< 0.001	255 ± 18	0.02	197 ± 45.3	0.33
	Seconds of immobility	19.8 ± 5.9	24.5 ± 8.9	0.31	35 ± 10	0.013	11.8 ± 6.3	0.04	31.7 ± 5.39	0.005

* p-value was calculated by comparing all the test values to the control group. *p < 0.05 was considered significant, SD=Standard deviation

#### Tail suspension test

After recording the duration of mobility and immobility, the average of the three readings was calculated. The results are shown in Table-1.

Rats treated with V and Hg took noticeably longer to reach the target quadrant in the Morris water maze (Table-1). Rats treated with V, Hg, or Cd remained in the original quadrant for a significantly longer period of time than rats treated with As. Rats treated with V, As, Hg, and Cd spent a notably longer amount of time in the target quadrant. In contrast to the control group, the As group demonstrated significantly greater time of mobility during the tail suspension test, whereas the V- and Cd-treated rats demonstrated significantly less time of mobility. Rats treated with Cd and Hg were immobile for noticeably longer periods of time than rats treated with As, who were significantly less immobile.

### Physiological tests

The intake of food and water was monitored throughout the 13 weeks and is listed in Table-2.

**Table-2:** Amount of urine (mL) and feces (gram) excreted by the rats when subjected to a metabolic cage for 12 h.

Physiological tests	Timeline	Control	Vanadium		Cadmium		Mercury		Arsenic	
				
		Mean ± SD	Mean ± SD	p-value	Mean ± SD	p-value	Mean ± SD	p-value	Mean ± SD	p-value
Food intake (g)	13^th^ week	30.9 ± 3.07	26.5 ± 2.20	< 0.001	33 ± 4.2	0.05	36 ± 6.3	< 0.001	30.3 ± 2.9	0.434
Water intake (mL)	13^th^ week	29.8 ± 4.09	19.16 ± 4.29	< 0.001	24.1 ± 2.21	< 0.001	29.2 ± 4.29	0.56	23.5 ± 2.72	< 0.001
Weight of the rats (g)	13^th^ week	166 ± 46.1	117 ± 10.8	0.001*	183 ± 61	0.423	205 ± 67.5	0.096	167 ± 36.1	0.933
Amount of urine (mL)	12^th^ week	12.5 ± 0.81	5.58 ± 1.63	< 0.001	12.7 ± 2.73	0.86	12.7 ± 1.55	0.76	12.3 ± 1.51	0.781
Amount of feces (g)	12^th^ week	10.9 ± 1.16	7.10 ± 1.35	< 0.001	9.33 ± 3.08	0.28	11.4 ± 2.26	0.62	9.47 ± 1.86	0.3

* p-value was calculated by comparing all the test values to the control group. *p < 0.05 was considered significant, SD=Standard deviation

#### Weight of the rats

The weight of the rats was monitored through 13 weeks and is presented in Table-2.

#### Amount of urine and feces

Urine and feces excreted by rats receiving heavy metal treatments were quantified using metabolic cages in the 12^th^ week of the study. Values were recorded and presented in Table-2.

Table-2 indicates that the food intake of the Cd- and V-treated groups was significantly lower than that of the control group, Hg, but the Cd-treated group consumed significantly more food than the control group. Over the course of 13 weeks, the water intake of the V, Cd, and As-treated rats was significantly lower than that of the control group. Over the course of 13 weeks, the weight of the V-treated rats was notably lower than that of the control rats. An analysis conducted between the V-treated rats and the control group revealed a noteworthy reduction in the amount of urine and feces that were collected.

### Biochemical investigations

#### Blood glucose estimation

The estimation was performed by providing the rats with an overnight fast of food on the 95^th^ day by puncturing the tail vein. The blood glucose ranges observed in this study are presented in Table-3.

**Table-3:** Biochemical investigations on animal model treated with heavy metals.

Biochemical tests/Groups	Control	Vanadium		Cadmium		Arsenic		Mercury	
				
	Mean ± SD	Mean ± SD	p-value	Mean ± SD	p-value	Mean ± SD	p-value	Mean ± SD	p-value
Blood glucose (mg/dL)^#^	117 ± 15	129 ± 23	0.3	144 ± 36	0.12	196 ± 44	0.002*	188 ± 52	0.01
PSA (ng/mL)^#^	0.09 ± 0.05	3.05 ± 2.9	0.002	0.13 ± 0.01	0.1	1.96 ± 2.2	0.04	0.68 ± 0.6	0.06
CEA (ng/ml)	27.07 ± 2.1	32.7 ± 1.4	0.001	29.9 ± 7.7	0.404	31.20 ± 1.2	0.002	30.8 ± 2.76	0.026
TSH (ng/mL)	0.13 ± 0.01	0.10 ± 0.01	0.1	0.87 ± 0.27	0.005*	0.27 ± 0.2	0.06	1.9 ± 1.1	0.005
T3 (ng/mL)	7.85 ± 0.8	7.88 ± 0.8	1	7.3 ± 0.2	0.8	6.4 ± 0.26	0.4	0.13 ± 0.12	0.002
T4 (ng/mL)	190.2 ± 41	193.3 ± 50	0.5	261 ± 25.3	0.01	193.0 ± 56	1	47.53 ± 13.2	0.002

**p-value was calculated by comparing all the test values to the control group.

* any value <0.05 was considered significant.

# mg/dL=milligrams per deciliter, ^#^ng/mL=Nanograms per milliliter (Detection values). PSA=Prostate-specific antigen, CEA=Carcinoembryonic antigen, TSH=Thyroid-stimulating hormone, SD=Standard deviation

#### ELISA

The serum samples were analyzed for selected biochemical parameters, such as PSA, CEA, and thyroid hormone levels. The thyroid type was also analyzed. The recorded results are shown in Table-3.

In Table-3, when comparing the As and control groups, blood glucose estimation in rats showed a statistically significant increase in glucose. All other groups, except for V, had hyperglycemia. According to the PSA kit (Origin Diagnostics) analysis results, rats treated with As and V showed statistically significant differences (higher than control) when compared with the control groups. A clear trend was noted in the evaluation of CEA scores. Only rats treated with V, Hg, or As exhibited significantly higher levels. Significant variations were found when thyroid markers were examined. Rats treated with Cd and Hg showed significantly higher TSH levels than the control group. Rats treated with Hg showed different patterns for T3 and T4. T4 levels were significantly higher in Cd-treated rats as well. The type of thyroid was analyzed using thyroid function tests as per Beynon and Pinneri [25]. After analyzing the thyroid hormone tests, it was determined that the control group’s thyroid function was normal, whereas the rats treated with Hg and Cd had secondary hypothyroidism.

Together, these results provide a thorough understanding of the various effects of exposure to heavy metals on physiological systems, highlighting the necessity of ongoing research to identify the underlying mechanisms and guide the development of mitigation strategies for possible health risks.

## Discussion

### Cognitive function tests

#### Morris water maze test

The Morris water maze test is where rodents are expected to reach the target quadrant, and any deviations from this test suggest neurological deficits, cognitive function, and behavioral misadaptability [26]. Finding the escape platform took longer in the V- and Hg-treated groups than in the other groups, suggesting a significant deficit in local geographic orientation and memory retrieval. This is consistent with earlier research that suggested that these elements have a neurotoxic effect on cognition [27]. With the exception of the V, As, and Cd-treated groups, there were notable variations in the amount of time spent in the original quadrant, highlighting the effects on perceptive deficiency and changes in exploratory pattern. All experimental groups consistently spent less time in the target quadrant than the control group, indicating altered navigation strategies and failed memory consolidation. Reduced preference for the target quadrant is a sign of altered navigation strategies or problems with spatial memory consolidation [28]. Exposure to a heavy metal mixture impaired cognitive function and memory in rats, with sex-specific differences noted, suggesting potential metal-metal interactions and a need for further research to understand the combined neurological impact of heavy metal mixtures as per a study conducted by Selorm *et al*. [29]. Many variables, including oxidative stress from free radical formation, disruption of neurotransmitters, and stimulation of neuronal excitons, have been linked to the central nervous system’s susceptibility to heavy metal toxicity, resulting in damage to multiple brain regions [30]. The effects of heavy metals on memory processes may differ among species; hence, it may not be appropriate to generalize the findings. However, mice do not experience this negative effect [31]. Despite this, the observed differences in our study highlight the need for focused research on the neurobiological mechanisms underlying these behavioral changes.

#### Rat tail suspension test

The rat tail suspension test provides a consistent method for quantifying depressive-like behavior, which helps evaluate neurobehavioral responses in preclinical studies. The initial phase of active struggle (mobility phase) in response to the tail suspension stressor was severely affected in the Cd- and V-treated groups, indicating a depressive-like effect. Although Hg-treated rats also showed a similar trend, the results were not statistically significant. The opposite was observed in the As-treated rats, where the active struggle phase was longer than the control time. During the study, it was observed that these (As treated) rats were generally hyperactive in their cages compared with the other treatment groups. Muñoz *et al*. [32] showed an association between As and the development of attention-deficit hyperactivity disorder in children. In the 2^nd^ phase of the test, a short period of immobility is expected. The Cd, Hg (Significant), and V (Not significant) groups showed a longer period of immobility compared with the controls. Feng *et al*. [33] and Volchegorskii *et al*. [34] reported that the immobility phase of the tail suspension test is caused by an incapacity or reluctance to maintain effort. This is compared to clinical findings in which there is frequently no consistent effort in performing a task, which is reflected in significant psychomotor deficits. As with Phase 1 of active struggle, the As-treated groups showed a lower value of Phase 2 immobility, again signifying hyperactivity disorder.

### Physiological tests

V exposure is known to cause metabolic toxicity, as evidenced by reduced food and drink intake, lower body weight, and reduced fecal and urine excretion [35]. The changes noted are signs of systemic toxicity affecting different organ systems. Gastrointestinal discomfort, decreased kidney function, and behavioral changes have been documented after V exposure [36]. A significant increase in food intake was observed in the Cd- and Hg-treated groups and the associated increase in weight signifies metabolism-related regulatory systems due to heavy metal exposure. Given the pandemic rise in obesity and diabetes mellitus, it may be worthwhile to pursue the neurobiological effects, hormone signaling, and control of metabolic pathways involved in hunger and energy balance in Cd and Hg toxicity [37, 38], a common contaminant of packaged food. Food consumption by the As-treated group indicated that As exposure did not have a direct impact on hunger, but water consumption was significantly decreased. In line with this finding, the weight of the rats did not differ from that of the control groups. The significantly lower water intake in this group indicates nephrotoxic effects that may interfere with water and electrolyte balance. Thus, the study of heavy metals has different physiological disturbances in rats, which requires further detailed elucidation.

### Biochemical investigations

Earlier research has shown a possible connection between exposure to heavy metals and glucose dyshomeostasis [39]. All heavy metal-treated groups showed higher fasting glucose values compared with the control group; however, a statistical difference was obtained with the As- and Hg-treated groups only. Kirkley *et al*. [40] showed that As disrupts a number of biological functions, notably the metabolism of glucose. Studies on Hg have shown contradictory results in which Hg exposure either caused hyperglycemia or showed no effect [41, 42]. Hence, each heavy metal may have varying effects on biological functions in general and glucose regulation and insulin sensitivity in particular. Similar conflicting results have been reported for Cd and V toxicity [42–45].

PSA, CEA, T3, T4, and TSH were selected to analyze the possible influence of heavy metals on the most sensitive tissues and those with high turnover.

Serum CEA levels are frequently used to measure the effectiveness of treatments and to modify the adhesion, spreading, proliferation, and migration of endothelial cells both *in vitro* and *in vivo* [46]. With the exception of Cd, all heavy metal-treated groups (V, Hg, As) showed significantly higher CEA levels than the controls. Similar results were found for PSA, whereas V showed significant elevation in PSA levels. Belonging to the Kallikrein family, PSA, thought to be expressed only in prostatic tissue, is now known to show tissue distribution in the breast, cervix, placenta, lungs, heart, etc. Its status as a tumor marker of the prostate gland has also changed over time because infection, inflammation, injury, or malignancies in the tissues where it is distributed can lead to a rise in PSA levels [47]. This calls for a thorough investigation of the molecular pathways that heavy metals use to induce CEA and PSA expression, as this may shed light on their possible involvement in acute and chronic toxicity [48, 49].

Thyroid tissues are by far very sensitive to changes in toxic elements, reactive oxygen species, and metabolic abnormalities. The effects of heavy metal poisoning on the thyroid gland are well documented. In this study, we found that Cd and Hg had serious and demonstrably harmful effects on the thyroid gland. Cd-treated rats showed secondary hypothyroidism, indicating pituitary involvement. Pamphlett *et al*. [50] stated that thyroid function abnormalities have been linked to Cd and Hg, which may cause hypothyroidism in people. These metals can cause inflammatory reactions, hinder the thyroid gland’s ability to absorb iodine, and interfere with the synthesis of thyroid hormones, all of which can lead to inflammation and damage to thyroid tissue. However, the most frequent reports indicate a picture of primary hypothyroidism in which the thyroid gland is affected [51]. A significant hypothyroid status with increased TSH and decreased T3 and T4 levels was observed in the Hg-treated groups. The incidences of autoimmune thyroiditis and thyroid cancer were previously reported by Boi *et al*. [52]. The mechanism is attributed to genotoxic effects, oxidative damage, and the autoimmune response. The thyroid function tests in the V- and As-treated groups were comparable to those of the control group.

The primary limitation of this study is the lack of investigation into molecular mechanisms within specific tissues, highlighting the need for future research to comprehensively understand the widespread effects of heavy metals. Further studies focusing on select heavy metals and their impact on specific molecular mechanistic pathways, organs, and their respective biomarkers will elucidate the nuances of heavy metal toxicity and their mechanisms of action.

## Conclusion

The evaluation revealed the complex and material-specific effects of heavy metal exposure on various physiological parameters in Wistar rats. Our study shed light on the effects and behavioral responses, cognitive processes, and quantitative evaluations of metabolic parameters. The noted changes in biochemical estimations highlight their extensive influence on endocrine and metabolic processes. The results of the study can be extrapolated to human health from exposure to environmental and occupational heavy metals. The investigation also necessitates further understanding of the molecular mechanisms underlying these complex effects, focusing on the reduction of health hazards and successful public health initiatives.

## Authors’ Contributions

SM: Data curation and formal analysis, PAM: Methodology. RMS: Project administration. SM, PAM, SH, HK: Original draft. YLR, AS, PAM, SM, and SH: Supervision and writing – review and editing of the manuscript. All authors have read, reviewed, and approved the final manuscript.

## References

[1] Sable H, Singh V, Kumar V (2024). Toxicological and bioremediation profiling of nonessential heavy metals (mercury, chromium, cadmium, aluminium) and their impact on human health:A review. Toxicol. Analyt. Clin.

[2] Moskalyk R.R, Alfantazi A.M (2003). Processing of vanadium:A review. Miner. Eng.

[3] Conti M.E (2006). Heavy Metals in Food Packaging's. Mineral Components in Foods.

[4] Grund S.C, Hanusch K, Wolf H.U (2011). Arsenic and arsenic compounds. IARC Monogr. Eval. Carcinog. Risks Hum.

[5] Balali-Mood M, Naseri K, Tahergorabi Z, Khazdair M.R, Sadeghi M (2021). Toxic mechanisms of five heavy metals:Mercury, lead, chromium, cadmium, and arsenic. Front. Pharmacol.

[6] Poças F, Oliveira J.C, Pereira J.R, Brandsch R (2011). Modelling migration from paper into a food simulant. Food Control.

[7] Jaishankar M, Tseten T, Anbalagan N, Mathew B.B, Beeregowda K.N (2014). Toxicity, mechanism and health effects of some heavy metals. Interdiscip. Toxicol.

[8] Imtiaz M, Rizwan M.S, Xiong S, Li H, Ashraf M, Shahzad S.M, Shahzad M, Rizwan M, Tu S (2015). Vanadium, recent advancements and research prospects:A review. Environ. Int.

[9] Huff J, Lunn R.M, Waalkes M.P, Tomatis L, Infante P.F (2007). Cadmium-induced cancers in animals and in humans. Int. J. Occup. Environ. Health.

[10] Spungen J.H (2019). Children's exposures to lead and cadmium:FDA total diet study 2014–16. Food Addit. Contam. Part A Chem. Anal. Control. Expo. Risk Assess.

[11] Pant R, Mathpal N, Chauhan R, Singh A, Gupta A (2024). A Review of Mercury Contamination in Water and Its Impact on Public Health. Springer Nature, Switzerland.

[12] Smith A.H, Arroyo A.P, Mazumder D.N, Kosnett M.J, Hernandez A.L, Beeris M, Smith M.M, Moore L.E (2000). Arsenic-induced skin lesions among Atacameño people in Northern Chile despite good nutrition and centuries of exposure. Environ. Health Perspect.

[13] Munera-Picazo S, Cano-Lamadrid M, Castaño-Iglesias M.C, Burló F, Carbonell-Barrachina A.A (2015). Arsenic in your food:Potential health hazards from arsenic found in rice. Nutr. Diet. Suppl.

[14] Tchounwou P.B, Yedjou C.G, Patlolla A.K, Sutton D.J (2012). Heavy metal toxicity and the environment. Mol. Clin. Environ. Toxicol.

[15] Mukhi S, Rukmini M.S, Manjrekar P.A, Iyyaswami R, Sindhu H (2024). Assessment of arsenic, vanadium, mercury, and cadmium in food and drug packaging. F1000Research.

[16] Percie du Sert N, Hurst V, Ahluwalia A, Alam S, Avey M.T, Baker M, Browne W.J, Clark A, Cuthill I.C, Dirnagl U, Emerson M, Garner P, Holgate S.T, Howells D.W, Karp N.A, Lazic S.E, Lidster K, MacCallum C.J, Macleod M, Pearl E.J, Petersen O.H, Rawle F, Reynolds P, Rooney K, Sena E.S, Silberberg S.D, Steckler T, Würbel H (2020). The ARRIVE guidelines 2.0. PLoS Biol.

[17] Suárez C, Guevara C.A (2022). CPCSEA guidelines for laboratory animal. J. Vet. Med. Health.

[18] Test No. 425 Acute Oral Toxicity:Up-and-Down Procedure-en-OECD.

[19] OECD (2022). Test No. 425. Acute Oral Toxicity:Up-and-Down Procedure OECD Publishing, Paris.

[20] Vangalapati B, Manjrekar P.A, Hegde A, Kumar A (2016). *Pterocarpus marsupium* heartwood extract restores learning, memory and cognitive flexibility in a STZ-NA induced diabetes animal model. Int. J. Pharm. Pharm. Sci.

[21] Zhai S.Y, Gu H.W, Wang C, Li Y.S, Tang H.B (2024). *Gynura procumbens* and selected metabolites:Amelioration of depressive-like behaviors in mice and risperidone-induced hyperprolactinemia in rats. Biomed. Pharmacother.

[22] Giral M, Armengol C, Gavaldà A (2022). Physiologic effects of housing rats in metabolic cages. Comp. Med.

[23] Tariq R.A, Al-Sudani B.T, Arif I.S (2020). Promising preclinical data to a possible antidiabetic drug. J. Int. Pharm. Res.

[24] Leary S, Johnson C.L (2020). AVMA Guidelines for the Euthanasia of Animals.

[25] Beynon M.E, Pinneri K (2016). An overview of the thyroid gland and thyroid-related deaths for the forensic pathologist. Acad. Forensic Pathol.

[26] Hadipour M, Refahi S, Jangravi Z, Meftahi G.H (2023). Tarooneh extract relieves anxiety-like behaviors and cognitive deficits by inhibiting synaptic loss in the hippocampus and frontal cortex in rats subjected to chronic restraint stress. 3 Biotech.

[27] D'Hooge R, De Deyn P.P (2001). Applications of the Morris water maze in the study of learning and memory. Brain Res. Brain Res. Rev.

[28] Bromley-Brits K, Deng Y, Song W (2011). Morris water maze test for learning and memory deficits in Alzheimer's disease model mice. J. Vis. Exp.

[29] Selorm S, Kenston F, Su H, Li Z, Kong L, Wang Y, Song X, Gu Y, Barber T, Aldinger J, Hua Q, Li Z, Ding M, Zhao J, Lin X (2018). The systemic toxicity of heavy metal mixtures in rats. Toxicol. Res. (Camb).

[30] Leelee Famii Z, Seleke-Ere Favour B (2019). Morris water maze test on neuroprotective effects of ethanolic extract of oyster mushroom (*Pleurotus ostreatus*) against neurotoxicity of mercury chloride in albino rats (*Rattus norvegicus*). World J. Adv. Res. Rev.

[31] Lu J, Zheng Y.L, Wu D.M, Sun D.X, Shan Q, Fan S.H (2007). Trace amounts of copper induce neurotoxicity in the cholesterol-fed mice through apoptosis. FEBS Lett.

[32] Muñoz M.P, Rubilar P, Valdés M, Muñoz-Quezada M.T, Gómez A, Saavedra M (2020). Attention deficit hyperactivity disorder and its association with heavy metals in children from Northern Chile. Int. J. Hyg. Environ. Health.

[33] Feng J, Liu L, Yang X, Lu F, Zhang M, Wu X, Sun L (2023). Rat bone responses to hindlimb unloading-reloading:Composition, BMD and mechanical properties. Acta Astronaut.

[34] Volchegorskii I.A, Miroshnichenko I.Y, Sinitskii A.I, Grobovoi S.I, Rassokhina L.M, Grobovoi S.I (2024). Effect of thioxypine and its components on behavioral despair in potential antidepressant screening tests. Pharm. Chem. J.

[35] Treviño S, Díaz A, Sánchez-Lara E, Sanchez-Gaytan B.L, Perez-Aguilar J.M, González-Vergara E (2019). Vanadium in biological action:Chemical, pharmacological aspects, and metabolic implications in diabetes mellitus. Biol. Trace Elem. Res.

[36] Bankole A, Ogunkeyede A, Adedosu T, Udeochu U, Agboro H, Isukuru E (2024). Assessment of heavy metal exposure in soils of Ihwrekreka communities, Delta State, Nigeria. J. Geosci. Environ. Prot.

[37] Shen T, Zhong L, Ji G, Chen B, Liao M, Li L, Huang H, Li J, Wei Y, Wu S, Chen Z, Ma W, Dong M, Wu B, Liu T, Chen Q (2024). Associations between metal (loid) exposure with overweight and obesity and abdominal obesity in the general population:A cross-sectional study in China. Chemosphere.

[38] Jeong S, Choi Y.J (2024). Investigating the influence of heavy metals and environmental factors on metabolic syndrome risk based on nutrient intake:Machine learning analysis of data from the eighth Korea national health and nutrition examination survey (KNHANES). Nutrients.

[39] Mostafalou S, Baeeri M, Bahadar H, Soltany-Rezaee-Rad M, Gholami M, Abdollahi M (2015). Molecular mechanisms involved in lead induced disruption of hepatic and pancreatic glucose metabolism. Environ. Toxicol. Pharmacol.

[40] Kirkley A.G, Carmean C.M, Ruiz D, Ye H, Regnier S.M, Poudel A, Hara M, Kamau W, Johnson D.N, Roberts A.A, Parsons P.J, Seino S, Sargis R.M (2018). Arsenic exposure induces glucose intolerance and alters global energy metabolism. Am. J. Physiol. Regul. Integr. Comp. Physiol.

[41] Guo Y, Lv Y, Liu X, Wang G (2023). Association between heavy metal mercury in body fluids and tissues and diabetes mellitus:A systematic review and meta-analysis. Ann. Transl. Med.

[42] Roy C, Tremblay P.Y, Ayotte P (2017). Is mercury exposure causing diabetes, metabolic syndrome and insulin resistance?A systematic review of the literature. Environ. Res.

[43] Saleh Al-Sowayan N, Mohammad AL-Sallali R (2023). The effect of aloin in blood glucose and antioxidants in male albino rats with Streptozotocin-induced diabetic. J. King Saud Univ. Sci.

[44] Buha A, Đukić-Ćosić D, Ćurčić M, Bulat Z, Antonijević B, Moulis J.M, Goumenou M, Wallace D (2020). Emerging links between cadmium exposure and insulin resistance:Human, animal, and cell study data. Toxics.

[45] Ji J.H, Jin M.H, Kang J.H, Lee S.I, Lee S, Kim S.H, Oh S.Y (2021). Relationship between heavy metal exposure and type 2 diabetes:A large-scale retrospective cohort study using occupational health examinations. BMJ Open.

[46] Zi-Yang Y, Kaixun Z, Dongling L, Zhou Y, Chengbin Z, Jimei C, Caojin Z (2020). Carcinoembryonic antigen levels are increased with pulmonary output in pulmonary hypertension due to congenital heart disease. J. Int. Med. Res.

[47] David M.K, Leslie S.W (2022). Prostate specific antigen. In:StatPearls.

[48] Mortazavi S.M, Mozdarani H (2014). PSA, CA19-9 and CEA tumour markers in blood serum of inhabitants of Ramsar, Iran. J. Envir. Radioact.

[49] Abbasy L, Mohammadzadeh A, Hasanzadeh M, Razmi N (2020). Development of a reliable bioanalytical method based on prostate specific antigen trapping on the cavity of molecular imprinted polymer towards sensing of PSA using binding affinity of PSA-MIP receptor:A novel biosensor. J. Pharm. Biomed. Anal.

[50] Pamphlett R, Doble P.A, Bishop D.P (2021). Mercury in the human thyroid gland:Potential implications for thyroid cancer, autoimmune thyroiditis, and hypothyroidism. PLoS One.

[51] Buha A, Matovic V, Antonijevic B, Bulat Z, Curcic M, Renieri E.A, Tsatsakis A.M, Schweitzer A, Wallace D (2018). Overview of cadmium thyroid disrupting effects and mechanisms. Int. J. Mol. Sci.

[52] Boi F, Pani F, Mariotti S (2017). Thyroid autoimmunity and thyroid cancer:Review focused on cytological studies. Rev. Eur. Thyroid J.

